# Summary version of the Standards, Options and Recommendations for the management of adult patients with intracranial glioma (2002)

**DOI:** 10.1038/sj.bjc.6601087

**Published:** 2003-08-15

**Authors:** D Frappaz, O Chinot, A Bataillard, M Ben Hassel, L Capelle, S Chanalet, M Chatel, D Figarella-Branger, Y Guegan, J Guyotat, K Hoang-Xuan, E Jouanneau, F Keime-Guibert, C Laforêt, C Linassier, H Loiseau, J P Maire, P Menei, S Rousmans, M Sanson, M P Sunyach

**Affiliations:** 1 Centre Léon Bérard, Lyon, France; 2 CHU La Timone, Marseille, France; 3 FNCLCC, Paris, France; 4 Centre Eugène Marquis, Rennes, France; 5 CHU Pitié Salpêtrière, Paris, France; 6 CHRU Pasteur, Nice, France; 7 CHR Pontchaillou, Rennes, France; 8 Hôpital Neurocardiologique Pierre Wertheimer, Bron, France; 9 CHU Bretonneau, Tours, France; 10 CHU Pellegrin Tripode, Bordeaux, France; 11 Hôpital St André, Bordeaux, France; 12 CHU, Angers, France

**Keywords:** glioma, practice guidelines

Gliomas are a complex and heterogeneous group of primary brain tumours. Mortality due to brain tumour has risen from 0.5% of cancer mortality in 1950 to almost 2.0% in 1985. The low incidence of this disease has limited the amount of data available, particularly for treatment.

## OBJECTIVES

The objective is to define guidelines for the management of adult patients with glioma. The management of spinal, non-glial, hypophyseal, neuronal tumours and schwannomas and the management of childhood glioma are not covered in this document.

## METHODS

The general methodology used has already been described (Fervers *et al*, 2001). For this specific SOR, a multidisciplinary working group was set up by the French National Federation of Cancer Centres (Fédération Nationale des Centres de Lutte Contre le Cancer–FNCLCC) and the Association of French-speaking Neuro-oncologists (Association des Neuro-Oncologues d'Expression Française–ANOCEF) to review the best available evidence on the management of adult patients with glioma.

Medline® and Cancerlit® were searched using a specific strategy, for the period 1990–2000. In addition, the members of the working group provided references from their personal sources up to 2001. The majority of the articles thus identified were in English or French. Many of the articles identified reported results for heterogeneous populations of patients (adults and children, and types of glioma), and therefore, when possible, information had to be extracted for specific populations and tumours. However, as this was not possible in all publications, not all the available literature could be analysed.

Following the selection and critical appraisal of the articles, the working group produced a document with the proposed ‘Standards’, ‘Options’ and ‘Recommendations’ (SORs) for the management of adult patients with glioma, based on scientific evidence or expert agreement. The document was then peer-reviewed by independent experts, and their comments were integrated in the final version. When all the members of the working group agree, based on the best available evidence, that a procedure or intervention is beneficial, inappropriate, or harmful, it is classified as a ‘*Standard*’, and when the majority agree, it is classified as an ‘*Option*’ (Table 1

**Table 1:** Definition of ‘Standards, options and recommendations’

*Standards*	Procedures or treatments that are considered to be of benefit, inappropriate or harmful by unanimous decision, based on the best available evidence
	
*Options*	Procedures or treatments that are considered to be of benefit, inappropriate of harmful by a majority, based on the best available evidence
	
*Recommendations*	Additional information to enable the available options to be ranked using explicit criteria (e.g., survival, toxicity) with an indication of the level of evidence

). In the SORs, there can be several ‘*Options*’ for a given clinical situation. ‘*Recommendations*’ provide additional information that enable the available options to be ranked using explicit criteria (e.g. survival, toxicity) with an indication of the level of evidence. These recommendations thus help clinicians to select an appropriate option. Thus, clinicians can make choices for the management of patients using this information and taking into consideration local circumstances, skills, equipment, resources and/or patient preferences. The adaptation of the SOR to the local situation is allowable if the reason for the choice is sufficiently transparent and this is crucial for successful implementation. Inclusion of patients in clinical trials is an appropriate form of patient management in oncology and is recommended frequently within the SORs, particularly in situations where only weak evidence exists to support a procedure or an intervention.

The type of evidence underlying any *‘Standard’, ‘Option’* or ‘*Recommendation*’ is indicated using a classification developed by the FNCLCC based on previously published methods. The level of evidence depends not only on the type and quality of the studies reviewed, but also on the concordance of the results (Table 2

**Table 2:** Definition of level of evidence

*Level A*
There exists high standard meta-analysis or several high-standard randomised clinical trials that give consistent results

*Level B*
There exist good quality evidence from randomised trials (B1) or prospective or retrospective studies (B2). The results are consistent when considered together

*Level C*
The methodology of the available studies is weak or their results are not consistent when considered together

*Level D*
Either the scientific data do not exist or there is only a series of cases
*Expert agreement*
The data do not exist for the method concerned, but the experts are unanimous in their judgement

). When no clear scientific evidence exists, judgement is made according to the professional experience and consensus of the expert group (‘expert agreement’^1^), and this is validated by the peer-review process.

This summary version has been translated from the French summary version, which was based on the integral version that will be published on the internet (http://www.fnclcc.fr).

## INCIDENCE AND RISK FACTORS FOR INTRACRANIAL GLIOMA

An increased incidence of intracranial glioma has been reported (level of evidence: C). With the exception of phacomatosis, familial predisposition to these tumours has been reported in fewer than 5% of patients, and extensive family screening is therefore not warranted.

Exposure to nitrate derivatives has been identified as a risk factor, and this constitutes occupational illness (level of evidence: C). No clear relationship between exposure to mobile telephones and increased risk of brain tumours has been documented (level of evidence: C). Exposure to low-frequency electromagnetic fields is thought to be associated with an increased risk (level of evidence: C). A national register of primary brain tumours should be created (recommendation).

## DIAGNOSIS: HISTOLOGY, MOLECULAR BIOLOGY AND CYTOGENETICS

To avoid misclassification (generally lower grade), the surgeon should ensure that the samples are representative of the lesion, particularly of any area of contrast enhancement present (standard). The quality of the sample should be sufficient to allow histological diagnosis, that is, type of glioma and grade (standard), and also molecular-biological and cytogenetic investigations (option).

### Sample processing

Some techniques require specific processing, therefore the sample taken by the surgeon should be processed immediately by the pathologist (recommendation). For histological diagnosis, the sample should be fixed with a 10% formaldehyde or zinc formaldehyde solution before embedding in paraffin (standard). Alcohol–formaldehyde–acetic acid (AFA) can be used for fixation (option) and is recommended for some molecular-biological investigations. Smear samples can be prepared from fresh tissue for diagnosis (recommendation). If the tissue is to be used for research purposes, a smear sample can be used to confirm tumour involvement of the processed tissue. For electronic microscopy, fixation with a 2% glutaraldehyde solution is possible (option).

Sterile cell culture can be used for cytogenetic investigations (option). The sample can be immediately frozen in liquid nitrogen for molecular–biological investigations and for inclusion in a tumour tissue bank (option).

### Histological diagnosis

The 2000 World Health Organization (WHO) classification is the standard for diagnosis and histoprognostic grading of glioma. Other classification systems (e.g. Smith, Daumas–Duport) can be used to complement the type and grading of oligodendroglioma (option).

Diagnostic or prognostic immunohistochemistry (GFAP, Ki67, etc.) can be performed (option). A search for deletions of 1p and 19q chromosome should be undertaken, particularly in patients with oligodendroglial tumours (recommendation). Comparison of the results from histology and imaging can help to establish the diagnosis (option). Review of the histology by an expert committee is recommended in all difficult samples and in all clinical trials (recommendation).

## IMAGING

### Diagnosis

Preoperative imaging should be performed with and without intravenous contrast medium (standard). MRI should be used in preference to CT scanning (standard, expert agreement). Three-dimensional scans should be taken using the same technique (standard). T1-weighted (with and without contrast medium), T2-weighted MR images and/or fluid-attenuated inversion recovery (FLAIR) imaging should be undertaken (standard). MR images should be converted into a digital format on a numerical support system (e.g. CD) for possible subsequent dosimetric studies (recommendation).

This imaging can be combined with functional MR, MR diffusion imaging, MR perfusion studies and/or with proton MR spectroscopy (options).

In the setting of clinical trials, it is possible to perform a positron emission tomography (PET) scan or a single photon emission computed tomography (SPECT) scan (options).

### Post-therapeutic and follow-up imaging

After surgical removal, imaging can be used to assess any residual tumour (option). MRI should be used, if possible (recommendation, level of evidence: B2), within 72 h, with and without contrast medium (recommendation). MRI is preferable to CT-scanning for follow-up of disease progression (recommendation).

## TREATMENT MODALITIES

### Surgery

#### 

##### Histological confirmation

Histological confirmation of the diagnosis should be obtained, because neuroradiological investigations are not sufficiently specific (standard, expert agreement). In exceptional situations, for example, elderly patients with a deep-seated lesion, presenting a very poor systemic or neurological condition, the clinician may consider that the risk from biopsy outweighs the risk from misdiagnosis and decide not to perform biopsy (option, expert agreement). However, this should remain exceptional.

##### Surgery

Criteria for surgery include the patient's age, general health performance status, as well as investigations (anatomical/functional data, presumed tumour type) and the technical support for surgery available (expert agreement).

Tumour resection should be optimal, that is with margins as wide as possible, avoiding any major functional risks (standard, expert agreement).

Surgical excision is the best means to obtain tissue samples that are representative of the whole lesion and to reduce the mass effect, if present (standard).

The use of technical aids (preoperative functional MR, ultrasound aspiration, surgical microscope, neuro-navigation, intraoperative brain mapping) can optimise surgical resection (option, expert agreement).

A biopsy (stereotactic, open skull) can be performed when surgical excision is not planned (standard).

### Radiotherapy

#### 

##### External-beam radiotherapy

Irradiation should be targeted using conventional external-beam radiotherapy (standard, level of evidence: B1). The gross tumour volume, or GTV, corresponds to contrast-enhanced image or, after complete excision, to the edges of the operative cavity (standard, expert agreement). For heterogeneous tumours (with hypo-intense and hyper-intense regions), and for hypo-intense tumours on T1-weighted images, the hypo-intense tumour volume is included in the GTV (recommendation, expert agreement).

The clinical tumour volume, or CTV, should include a safety margin of 20 mm outside the GTV limit in all three dimensions (standard, expert agreement). This safety margin can be reduced, depending on the grade, histological type and the tumour volume, (option, expert agreement). Noncoplanar focalised multiple beam (3–5) should be used to minimise the total fractionated dose delivered to the non-diseased brain (standard). Dose–volume histograms can be useful for defining the best treatment plan (option).

All fields should be irradiated the same day with a fractionated dose varying from 1.8 to 2 Gy per fraction and per day, five times per week (standard). The dose should be adapted according to the histological type and the grade of the lesion and should not exceed a total of 60 Gy (standard).

Prophylactic corticosteroid treatment should not be prescribed routinely (option, expert agreement), but can be used to reduce the risk of acute or early–delayed encephalopathy (radiation-induced oedema).

##### Complications following external-beam radiotherapy

Clinical and/or radiological deterioration in the 2 months after the end of radiotherapy should be interpreted with caution and not automatically be considered as a treatment failure (standard, level of evidence: C). The irradiation protocol (volume, total dose and particularly the dose per fraction), the presence of risk factors such as age (over 50 years old) and/or previous vascular disease (hypertension, diabetes, hyperlipidaemia) are associated with an increased risk for late neurological complications (radionecrosis, radiation-induced leucoencephalopathy) (level of evidence: C).

The results from FDG-PET scanning, proton MR spectroscopy or ^99m^Tc methoxy isobutyl isonitrile (MIBI) brain scintigraphy can help the differential diagnosis between recurrence and radionecrosis (option).

##### Other irradiation modalities

Brachytherapy (level of evidence: B2), stereotactic radiotherapy (expert agreement), use of radio-potentiation (level of evidence: B1) and heavy particles (expert agreement) should only be used in the setting of clinical trials since their efficacy has not been proved (recommendation).

### Chemotherapy

#### 

##### Efficacy of systemic chemotherapy

The efficacy of the following nitrosourea derivatives has been reported: 1,3-bis(2-chloroethyl)-1- (BCNU) (level of evidence: A); 1-(2-chloroethyl)-3-cyclohexyl-1-nitrosourea (CCNU) (level of evidence: C) and fotemustine (level of evidence: C). Nitrosourea molecules, particularly carmustine, have only been evaluated in clinical trials using definitions of response that are no longer used.

Nitrosourea derivatives can be used in the PCV combination: procarbazine, CCNU and vincristine (option, level of evidence: B2).

For second-line treatment, temozolomide (level of evidence: variable depending on the histology of the tumour), platinum derivatives either as a mono- or poly-chemotherapy (level of evidence: C) or procarbazine (expert agreement) can be considered. It is also possible to use another nitrosourea, if the time between the first- and second-line treatment is sufficiently long (level of evidence: D).

##### Efficacy of local chemotherapy

A local implant of BCNU can be considered in patients with recurrent disease (option, level of evidence: C). Other regional drug delivery strategies should only be envisaged in the setting of clinical trials (recommendation).

### Biological treatments (currently being evaluated)

Pharmacological agents with specific modes of action (e.g. protein kinase C inhibitors) targeting with monoclonal antibodies or ligands, immunotherapy and/or gene therapy should only be used in the setting of clinical trials (recommendation).

### Concomitant medical treatments

#### 

##### Treatment of oedema

Patients with clinical or radiological evidence of brain oedema should be treated (standard, expert agreement). The minimal effective dose should be determined and regularly re-evaluated (standard, expert agreement).

Treatment with corticosteroid or, less frequently, an osmotic agent can be considered (option, expert agreement). Patients should be monitored for side effects (standard, expert agreement). Methylprednisolone and prednisolone should be prescribed if possible, as single daily doses, in the morning (recommendation, expert agreement). If lymphoma is suspected, corticosteroid therapy should be avoided prior to obtaining histological confirmation, except for those patients whose neurological status requires this therapy (standard, expert agreement).

The clinical and radiological evaluation of the tumour evolution should take into account the variations in the dose of corticosteroid (standard, expert agreement).

##### Preventive treatment for gastrointestinal complications

Perioperative H2-receptor blockers or proton pump inhibitors can be used to prevent gastrointestinal complications in patients receiving high doses of corticosteroids and/or in those with risk factors for ulcers (e.g. previous ulcers, concomitant anticoagulant or NSAID) (recommendation).

#### Treatment of epilepsy

Peri-operative treatment: Treatment for epilepsy can be prescribed routinely during the perioperative period for patients who have had seizures (standard). In other patient, peri-operative anticonvulsant treatment is an option (option, level of evidence: C).

Post-operative treatment: In patients with previous seizures, anticonvulsant treatment should be continued in the postoperative period (standard). Since the efficacy of antiepileptic treatment in patients who have not had seizures has not been demonstrated, its prescription should be tailored to each patient (option, expert agreement).

There are no data to guide the choice of which drug(s) should be used for antiepileptic treatment (recommendation). Any inducing and/or potentiating effect on the toxic effects of the chemotherapy should be taken into consideration (recommendation, expert agreement). First-line treatment should be single-drug treatment (recommendation).

##### Analgesic treatment

Appropriate analgesic treatment should be prescribed when necessary: for example, for intracranial hypertension, neoplastic meningitis, pain associated with retractions due to permanent deficit (standard).

##### Anticoagulant treatment

Surveillance, prevention and treatment for thromboembolism should be performed, since this occurs frequently in patients with glioma (standard). Prophylactic use of low-molecular weight heparin and compression stockings is recommended for preventing perioperative thromboembolic complications (recommendation, level of evidence: B2). After 4–5-days of surgery, in the event of a thromboembolic complication, anticoagulant treatment at a therapeutic dose can be prescribed, without undue haemorrhagic risk (recommendation, expert agreement).

## SPECIFIC TREATMENT STRATEGIES

The management of patients with suspected glioma should be discussed with a multidisciplinary neuro-oncology team (standard).

### Grade 3 and 4 glioma

#### 

##### Prognostic factors

Age and preoperative functional status, as well as histological type and grade of the tumour are recognised prognostic factors (level of evidence: A).

In patients with oligodendroglioma, deletion of chromosome 1p (particularly when there is also a deletion of chromosome 19q) is a favourable prognostic factor for survival and probably for treatment response (level of evidence: B2).

##### Diagnosis and initial treatment

For biopsy or surgery, all patients should be routinely transferred to a specialist centre (standard).

All patients, except those with a high physiological age, and/or those with co-morbidities and/or with a poor performance status, and/or with lesions in functional, multifocal or centrally localised zones, should be offered optimal surgical resection when technically feasible, and if there is a low risk of permanent postoperative functional deterioration (standard, expert agreement). If this is not possible, histological evidence should be obtained by biopsy (standard).

Investigations to assess residual tumour after surgical resection can be undertaken (see section on Post-therapeutic and follow-up imaging), but their prognostic value, in terms of survival, is controversial (option, expert agreement).

**Figure 1:**
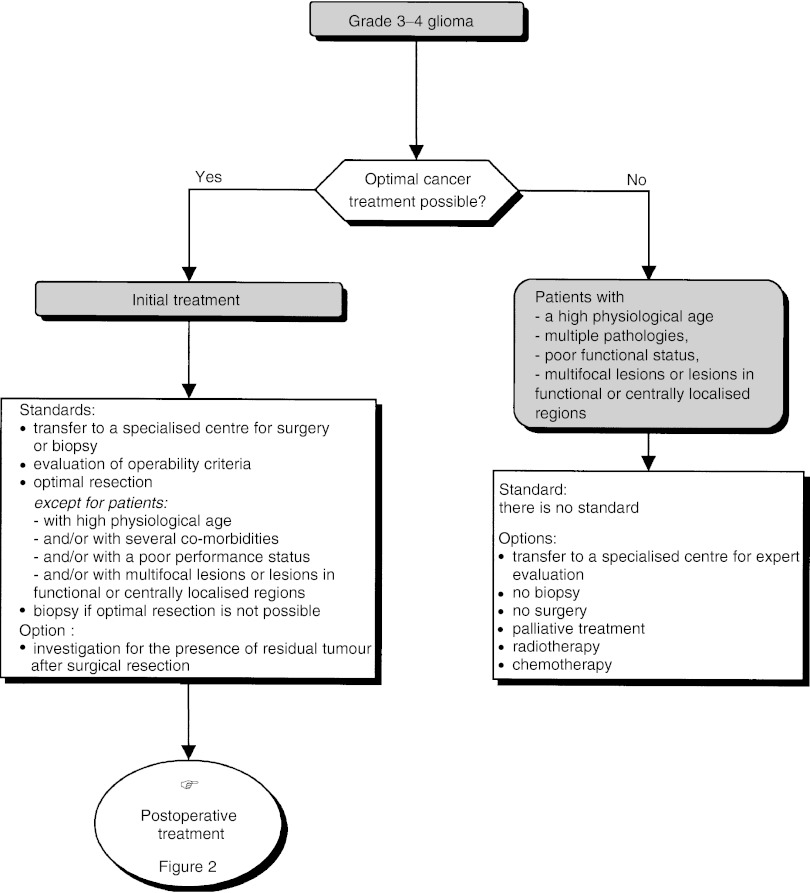
Supratentorial grade 3–4 glioma–initial management.

Optimal cancer treatment plans are not feasible in only a low percentage of patients (high physiological age, multiple pathologies, poor functional status, centrally localised lesion, etc.) and in this rare situation biopsy is not mandatory (option). In this situation, palliative treatment, radiotherapy or chemotherapy can be offered, tailored to the individual patient (options) (Figure 1

).

##### Postoperative treatment (Figure 2)

**Figure 2:**
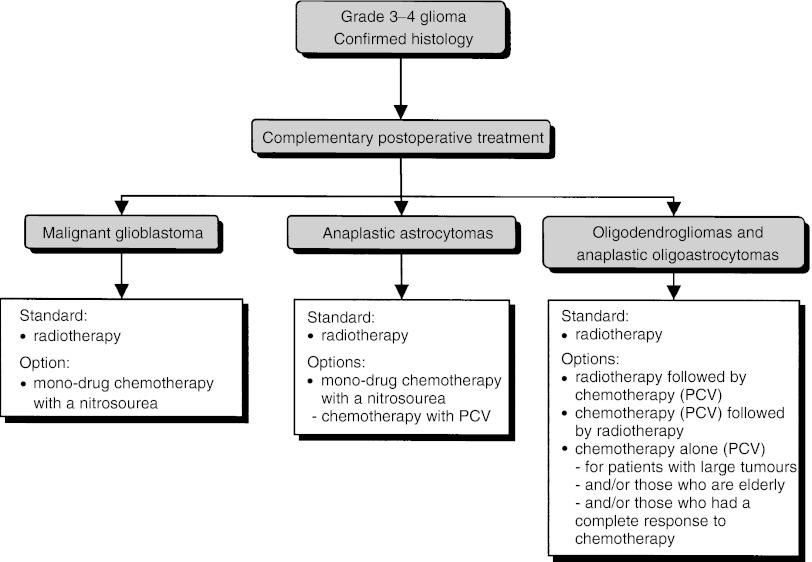
Supratentorial grade 3–4 glioma–postoperative treatment.

All histology and findings from imaging and clinical examinations should be taken into account before initiating any additional anticancer treatment to verify the coherence of the clinical picture (recommendation). Additional treatment should be started within a month (recommendation). The modalities of radiotherapy and chemotherapy should be adapted to the patient's status.

Patients should be included in clinical trials to evaluate postoperative treatment for high-grade glial tumours (recommendation).

First-line external-beam radiotherapy should be offered to patients with high-grade glioma since it has been shown to improve survival (standard, level of evidence: A) although these clinical trials excluded patients with poor prognostic factors, such as a low Karnofsky score and/or advanced physiological age and/or large or multifocal tumours. A total dose of 60 Gy should be delivered, with a fractionation from 1.8 to 2 Gy per fraction and per day (recommendation).

Glioblastoma and anaplastic astrocytoma: Radiotherapy should be offered (standard, level of evidence: A). This can be combined with chemotherapy with a nitrosourea-based chemotherapy (option, level of evidence: B1).

When chemotherapy is selected, mono-drug chemotherapy with a nitrosourea should be offered to patients with glioblastoma (standard, level of evidence: A) and either mono-drug chemotherapy with a nitrosourea (BCNU), or multidrug chemotherapy with procarbazine, lomustine, and vincristine (PCV) for patients with anaplastic astrocytoma (recommendation, level of evidence: C).

Anaplastic oligodendroglioma and oligoastrocytoma: Radiotherapy should be proposed for patients with anaplastic oligodendroglioma and oligoastrocytoma (standard, level of evidence: B2). Chemotherapy (PCV) has been shown to be efficacious in patients with oligodendroglioma. A combination of radiotherapy and chemotherapy can be considered, although the optimal timing of chemotherapy (neoadjuvant treatment *vs* adjuvant treatment *vs* treatment when the tumour recurs) has not been defined (option, level of evidence: B2). In selected patients with large unresectable tumours, and/or those who are elderly, and/or those who have had a complete response to neoadjuvant chemotherapy, it is not necessary to administer radiotherapy routinely (option, expert agreement).

##### Treatment of tumour recurrence (Figure 3)

**Figure 3:**
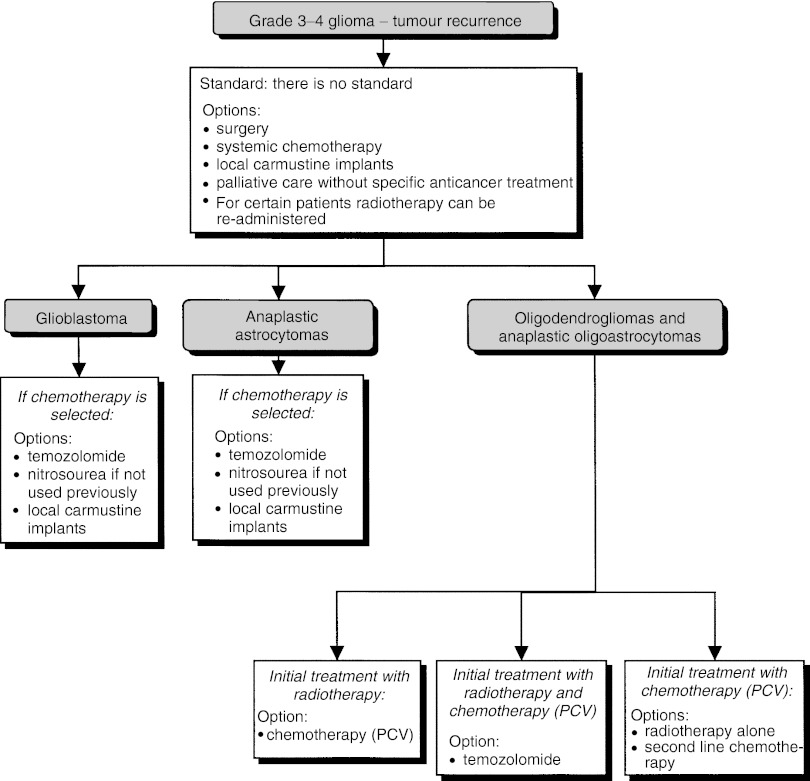
Supratentorial grade 3–4 glioma–treatment for tumour recurrence.

Patients should be included in clinical trials to evaluate treatment of tumour recurrence (recommendation).

Five therapeutic options can be considered (options, expert agreement): surgery, systemic chemotherapy, local chemotherapy, second-line radiotherapy or palliative care without specific anticancer treatment. The decision to perform surgery should only be taken after multidisciplinary consultation (recommendation). For selected patients, newer irradiation techniques can be considered, using different modalities (brachytherapy, stereotactic radiotherapy) (option).

Glioblastoma: There is no standard. Five therapeutic options can be considered (options, expert agreement): surgery, systemic chemotherapy, local chemotherapy, second-line radiotherapy or palliative care without specific anticancer treatment. The following drugs have shown moderate efficacy, and can be used if chemotherapy is indicated: temozolomide (option, level of evidence: C), nitrosourea molecules (option) and carmustine implants (option, level of evidence: C).

Anaplastic astrocytoma: There is no standard. Five therapeutic options can be envisaged (options, expert agreement): surgery, systemic chemotherapy, local chemotherapy, second-line radiotherapy or palliative care without specific anticancer treatment. Temozolomide, which has been shown to have significant efficacy (option, level of evidence: C), and carmustine implants, which have been shown to have moderate efficacy, can be administered if chemotherapy is indicated (option, level of evidence: C). Nitrosourea molecules can be proposed, if the patient has not received them previously (option, expert opinion).

Anaplastic oligodendroglioma and oligoastrocytoma: The second-line strategy depends on the first-line treatment used:

- In patients with recurrence after radiotherapy alone: chemotherapy with PCV can be considered (option, level of evidence: B2);
- In patients with recurrence after radiotherapy and chemotherapy with PCV: chemotherapy with temozolomide can be considered (option, level of evidence: C);
- In patients with recurrence after chemotherapy alone: radiotherapy should be considered when possible (option). If radiotherapy is not possible (older patients in poor condition with extensive tumour), second-line chemotherapy (option) can be considered.

### Grade 2 glioma (Figure 4)

**Figure 4:**
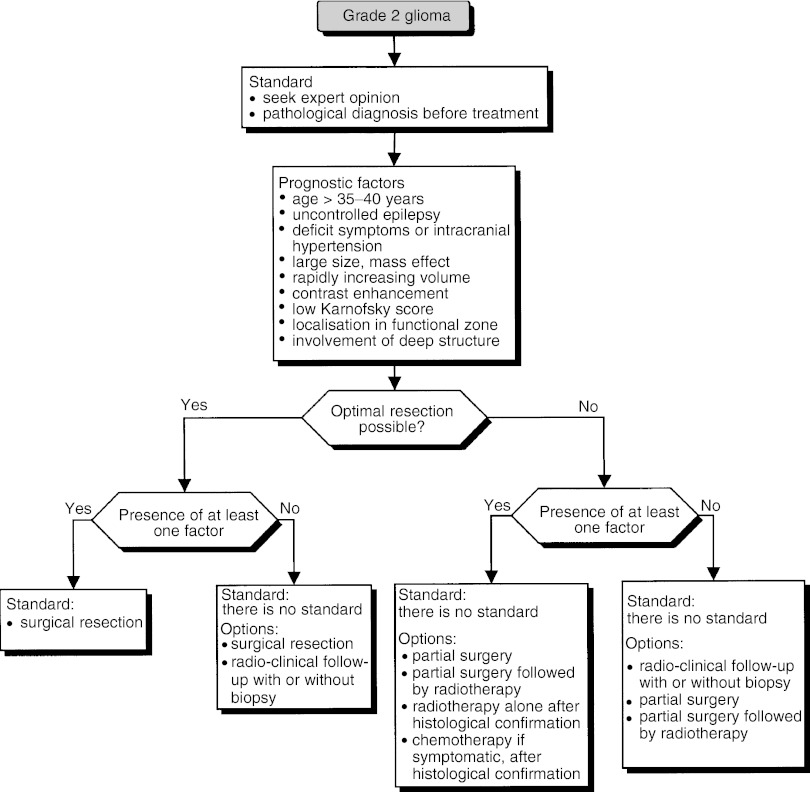
Grade 2 glioma.

The therapeutic decision must weigh the benefit of providing relief for distressing symptoms and avoiding or delaying anaplastic transformation, against the iatrogenic risk from treatment, particularly in patients whose tumour may fail to progress for a long time.

#### 

##### Prognostic factors

The factors for poor prognosis in patients with grade 2 glioma are:

- age ⩾35–40 years (level of evidence: B1),
- low Karnofsky score (level of evidence : B1),
- intracranial hypertension, functional deficit (level of evidence: C),
- uncontrolled epilepsy (level of evidence: C),
- large or rapidly increasing tumour volume and mass effect (level of evidence: C),
- localisation in a functional zone (level of evidence: C),
- involvement of deep structures (level of evidence: C),
- contrast enhancement on MR images (level of evidence: C).

##### Diagnosis and initial treatment

Radiological evaluation of grade 2 glioma should be based on MRI, both for diagnosis and follow-up (standard).

For patients with a grade 2 glioma, optimal resection involves a total or subtotal removal of the tumour volume defined in T2 and/or the FLAIR sequence on MRI (expert agreement).

The prognostic value of complete resection is uncertain, but when it is possible to aim for radiologically complete resection safely, surgery should be undertaken (standard, expert agreement). When radiotherapy is proposed, the dose should be between 45 and 54 Gy (standard, level of evidence: B2). It is recommended to use a dose between 50 and 54 Gy (recommendation, expert agreement). Chemotherapy can be proposed, in symptomatic oligodendroglial tumours, preferentially in clinical trials, since its role in this indication is uncertain (option, level of evidence: D).

The therapeutic strategy is based on the operability of the tumour and prognostic factors.

If optimal resection is possible:

- In the presence of at least one poor prognostic factor, surgical resection should be undertaken (standard).
- In the absence of poor prognostic factors, surgical resection or surveillance with or without biopsy can be considered (options).

If optimal resection is not possible:

- In the presence of at least one poor prognostic factor, partial resection, partial resection followed by radiotherapy, radiotherapy alone and chemotherapy can be proposed (options). The last two options should only be considered after histological confirmation.
- In the absence of poor prognostic factors, follow-up with or without biopsy, partial resection, partial resection followed by radiotherapy or biopsy followed by radiotherapy can be considered (options).

Patients should be included in clinical trials to evaluate therapeutic strategies (recommendation).

### Gliomatosis cerebri

The diagnosis of gliomatosis cerebri should be based on a comparison of the biopsy and radiological results (standard, expert agreement).

Three treatment options can be considered: chemotherapy alone (option, level of evidence: D); follow-up for asymptomatic patients, without radiological signs of progression (option); and whole-brain radiotherapy (option).

### Pilocytic astrocytoma, subependymoma and xanthoastrocytoma

#### 

##### Pilocytic astrocytoma (Figure 5)

**Figure 5:**
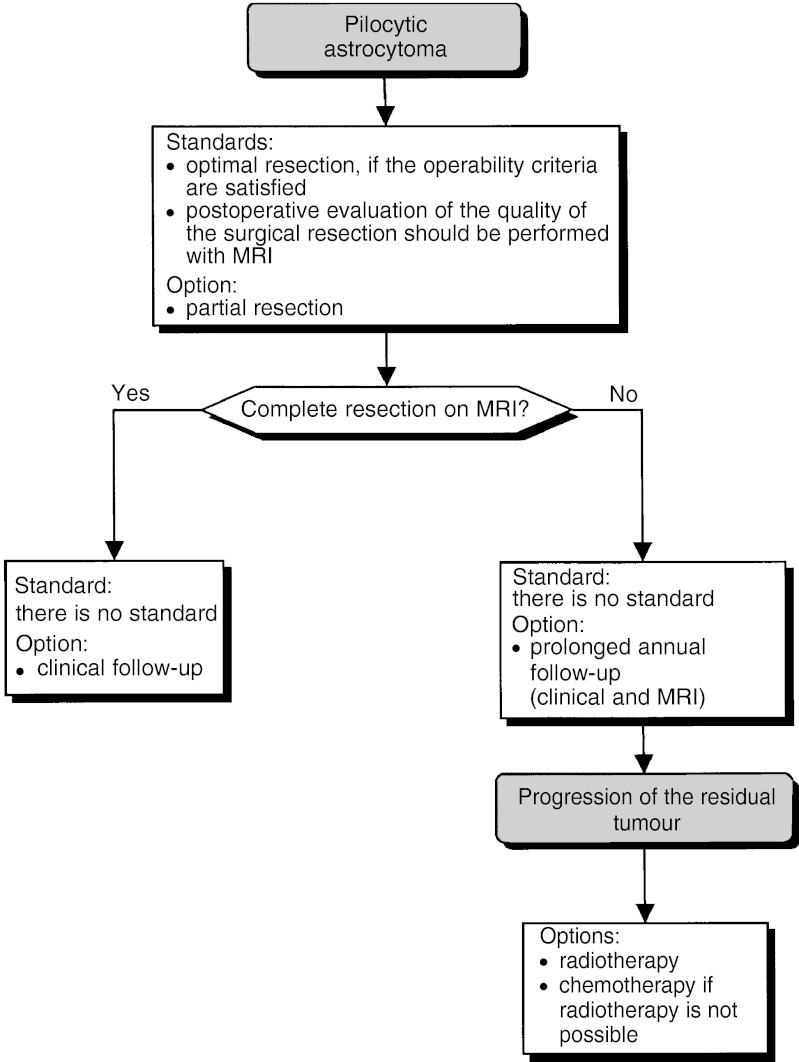
Pilocytic astrocytoma.

Pilocytic astrocytoma should no longer be called low-grade astrocytoma or glioma. The growth of pilocytic astrocytoma is slow, and sometimes absent, particularly for neurofibromatosis type 1.

Complete surgical resection significantly improves survival and often cures these patients (level of evidence: C). Optimal surgical resection should be offered to patients satisfying the operability criteria (standard). Even if complete resection is not possible, surgery can be considered, if the operability criteria are satisfied (option).

Postoperative evaluation of the quality of the surgical resection should be performed with MRI (standard, expert agreement). If the MRI confirms complete resection, simple clinical follow-up is indicated (option, expert agreement). If the resection is incomplete, annual follow-up, over many years (clinical and MRI), should be undertaken (option, expert agreement).

When resection is incomplete or not possible, and there is progression of the tumour, radiotherapy and/or chemotherapy can be considered although the indications and optimal modalities are uncertain (option).

##### Pleomorphic xanthoastrocytoma (Figure 6)

**Figure 6:**
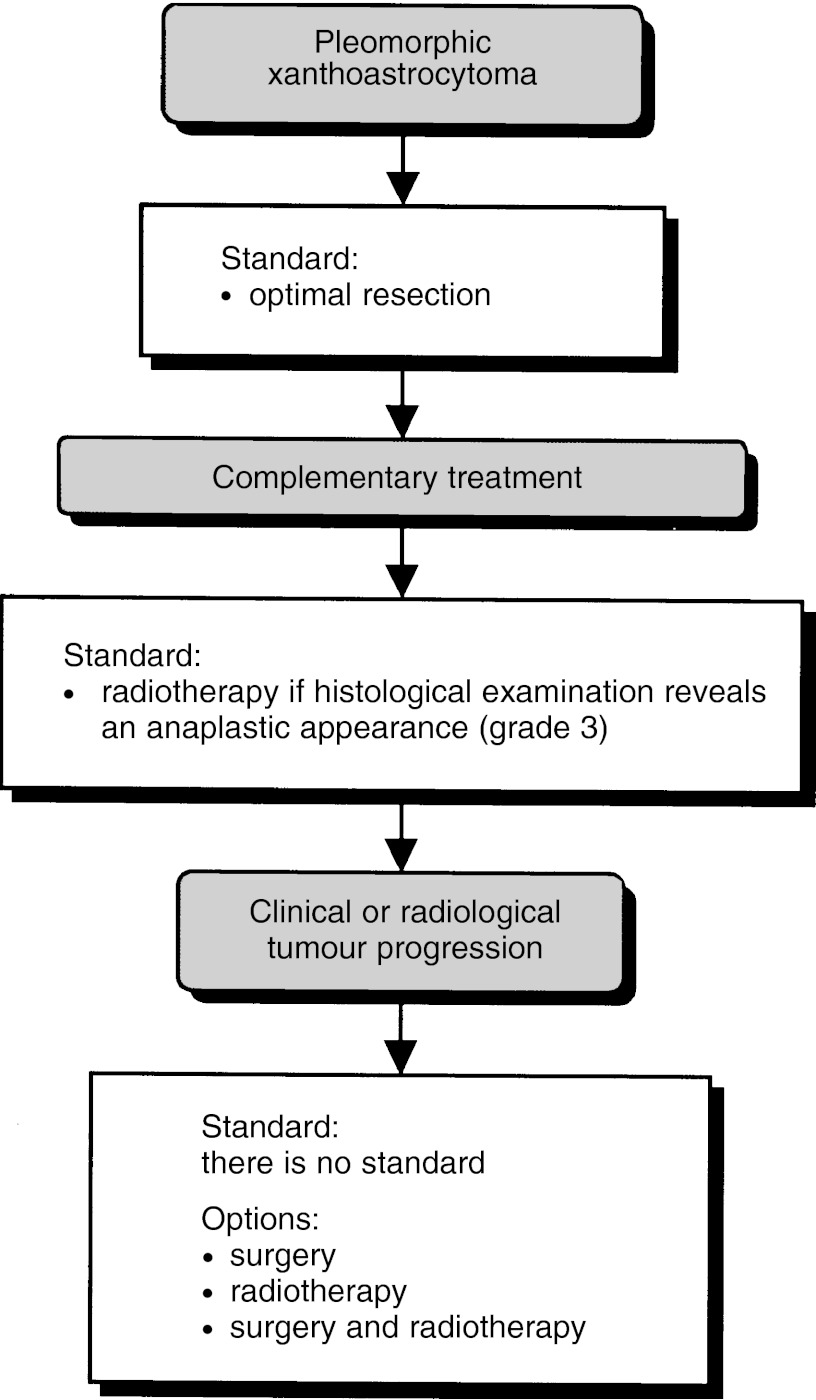
Pleomorphic xanthoastrocytoma.

Optimal surgical resection should be undertaken in all patients (standard, level of evidence: C).

If the histological examination reveals anaplastic change (grade 3), postoperative external-beam radiotherapy should be undertaken, irrespective of the quality of the surgical excision (standard, level of evidence: C).

If clinical or radiological tumour progression is observed, surgery and/or external-beam radiotherapy can be considered (option, level of evidence: C).

Clinical follow-up should include MRI (recommendation).

A pleomorphic xanthoastrocytoma register should be established (recommendation).

### Subependymoma (Figure 7)

**Figure 7:**
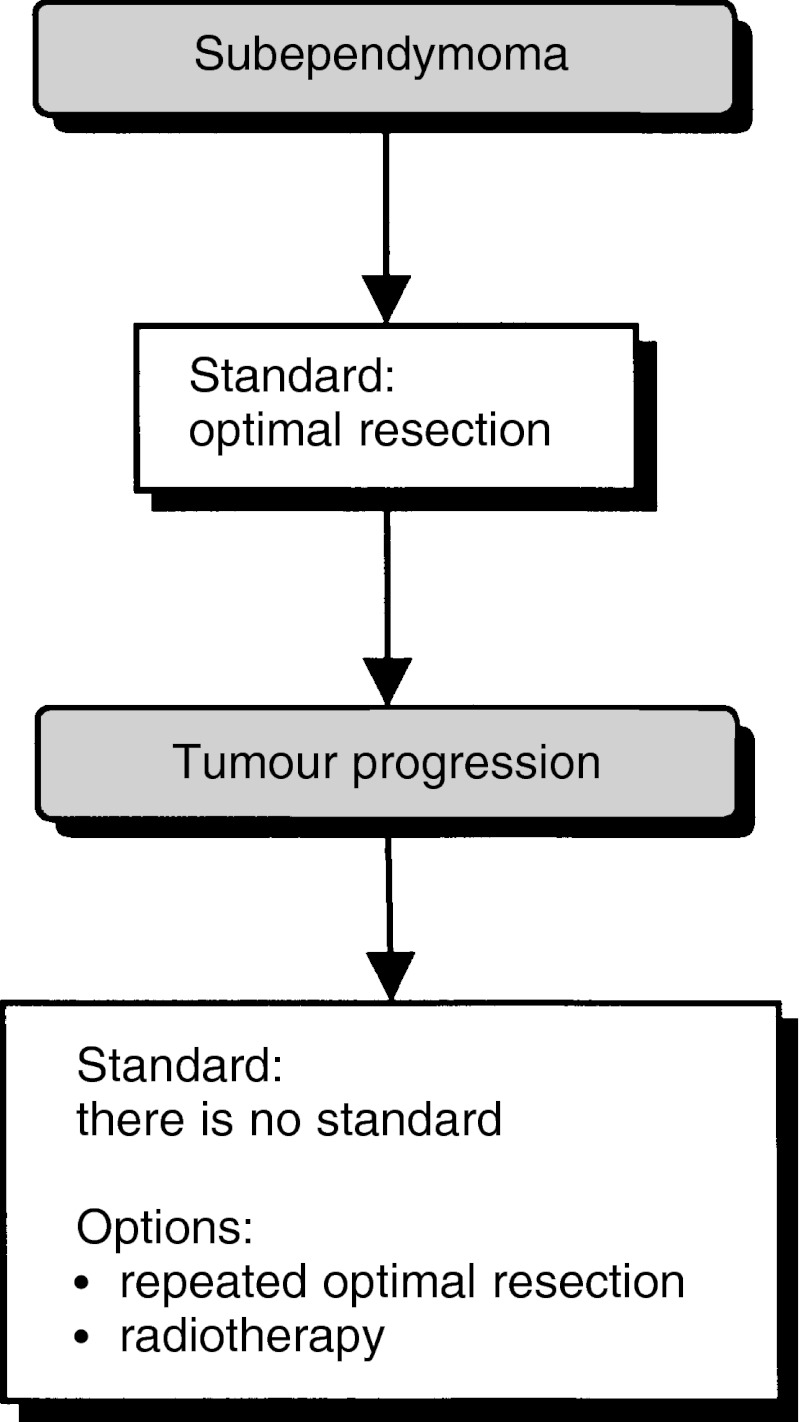
Subependymoma.

First-line treatment for a symptomatic subependymoma is optimal resection (standard). In patients with recurrence, further optimal resection or radiotherapy can be considered (option).

### Intracranial ependymoma (Figure 8)

**Figure 8:**
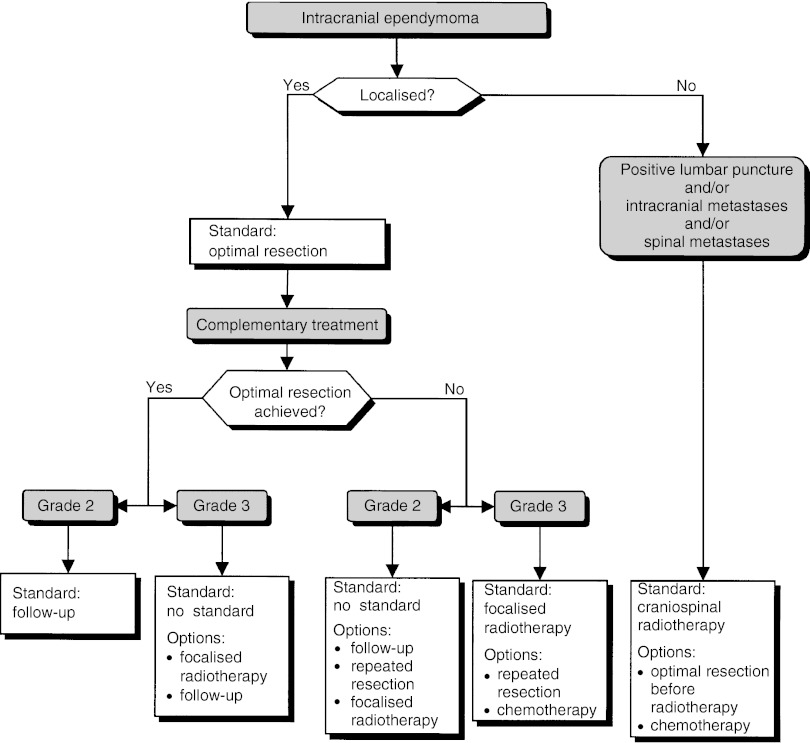
Intracranial ependymoma.

The histological grade can be taken into consideration, but it has an uncertain prognostic value (option). The work-up can include a spinal MRI and a lumbar puncture in patients with infratentorial tumours (option).

#### 

##### Localised lesions

Surgery is the standard treatment (standard, level of evidence: B) and complete surgical resection confirmed by early postoperative MRI is a good prognostic factor (level of evidence: B2). If radiotherapy is administered, localised radiotherapy, not craniospinal radiotherapy, should be proposed (standard, expert agreement).

In patients with complete resection and a grade 2 tumour, no complementary treatment is necessary (standard). For patients with a grade 3 tumour, focalised postoperative radiotherapy (option, level of evidence: C) or follow-up (option) can be offered.

In patients with incomplete resection and a grade 2 tumour, the treatment options are: follow-up, further resection or localised postoperative radiotherapy (options, level of evidence: C). If the tumour is grade 3, localised radiotherapy should be offered (standard). In this case, further resection or chemotherapy can be considered (options).

In patients with recurrence, further resection, radiotherapy, chemotherapy or palliative treatment can be considered (options).

##### Metastatic lesions

Patients with metastatic disease at presentation have a poor prognosis (level of evidence: B2).

Craniospinal radiotherapy should be offered (standard, expert agreement). Optimal surgical resection can be undertaken before radiotherapy (option). Chemotherapy can be offered (option).

### Brain stem glioma (Figure 9)

**Figure 9:**
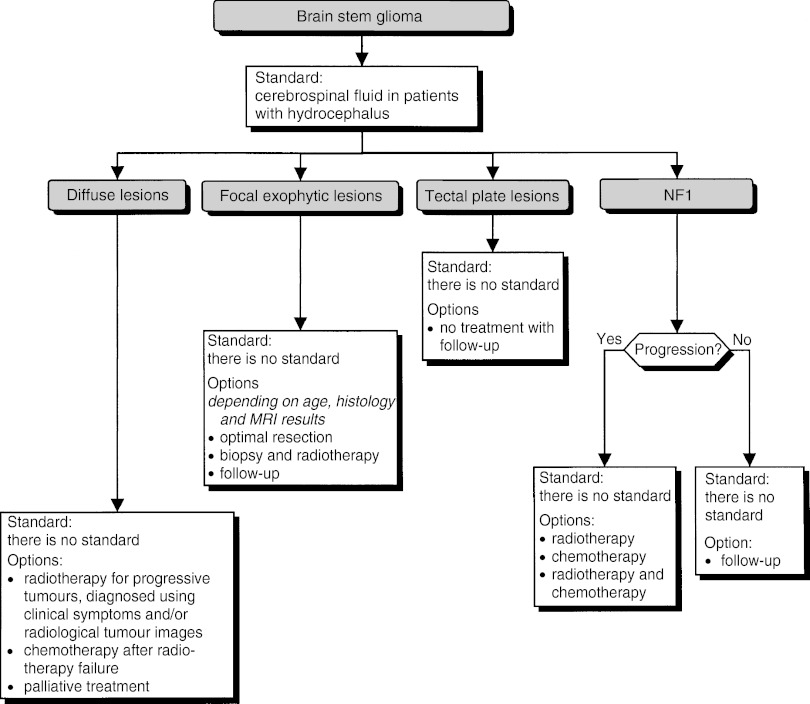
Brain stem glioma.

In patients with hydrocephalus, a cerebrospinal fluid shunt should be offered (standard). Lesions with different prognoses can be identified from their localisation and their neuro-radiological appearance.

#### 

##### Diffuse lesions

Biopsy can be considered (option). The patient's clinical status should be considered in any treatment decision (recommendation):

- Only progressive tumours, as diagnosed from clinical symptoms and/or radiological images, should be treated with radiotherapy (option, level of evidence: C).
- Chemotherapy can be considered after failure of radiotherapy (option).
- For certain patients, palliative treatment only can be considered (option).

##### Focal exophytic lesions

Biopsy or resection can be considered (option). Depending on the histology (if this can be determined: see section on each histological type), age and MRI results, optimal resection, radiotherapy or follow-up alone can be considered (options). An optimal resection should be undertaken whenever possible (recommendation).

##### Tectal plate lesions

No treatment, with follow-up alone, can be considered (option).

##### Neurofibromatosis type 1

Follow-up alone can be considered for patients with non-progressive brain stem glioma (option). Radiotherapy and/or chemotherapy can be considered for patients with progressive brain stem glioma (option).

## Appendix

A Adams (CHU Kremlin Bicêtre, Kremlin Bicêtre, France) C Alapetite (Institut Curie, Paris, France), A Ameri (CH, Meaux, France), O Arsene (CH, Blois, France), JJ Auregan (Centre Guillaume de Varye, Saint-Doulchard, France), A Autret (CHU Bretonneau, Tours, France), N Barbet (Centre d'Oncologie Radiothérapie, Macon, France), P Beauchene (CH Bellevue, Saint-Etienne, France), L Benboubker (CHU, Tours, France), J Boulliat (CH, Bourg-en-Bresse, France), H Boissonnet (Fondation Rothschild, Paris, France), H Bourgeois (CHU, Poitiers, France), JL Brault (Hôpital de Fontenoy, Chartres, France), P Bret (Hôpital Neurocardiologique Pierre Wertheimer, Bron, France), J Brunon (Clinique Mutualiste, Saint Etienne, France), B Bui (Institut Bergonié, Bordeaux, France), G Calais (CHU, Tours, France), H Cambon (CH, Saint Germain en Laye, France), C Campello (CHU, Nîmes, France), PE Cailleux (Clinique Fleming, Tours, France), A Carpentier (CHU Pitié-Salpêtrière, Paris, France), JM Caudrelier (Centre Oscar Lambret, Lille, France), D Celerier (Clinique Paulmy, Bayonne, France), J Chalençon (Centre Léon Bérard, Lyon, France), B Chassande (Etampes, France), N Cheron (Hôpital de Bligny, Briis sous Forges, France), L Cinotti (Hôpital Neurocardiologique Pierre Wertheimer, Bron, France), P Colin (Polyclinique Courlancy, Reims, France), P Cornu (CHU Pitié-Salpêtrière, Paris, France), D Cote (CH, Fontainebleau, France), JP Cottier (CHU, Tours, France), P Deck (CHU Mondor, Créteil, France), JY Delattre (CHU Pitié-Salpêtrière, Paris, France), D Denvil (CHU Pitié-salpêtrière, Paris, France), JM Derlon (CHRU, Caen, France), R Deruty (Hôpital Neurocardiologique Pierre Wertheimer, Bron, France), G Desfosse (Hôpital de la Cité Universitaire, Paris, France), C Desuzingues (Centre Léon Bérard, Lyon, France), Y Devaux (Centre Léon Bérard, Lyon, France), E Diot (CH Lucien Hussel, Vienne, France), F Dubois (CHU Roger Salengro, Lille, France), H Duffau (CHU Pitié-Salpêtrière, Paris, France), E Ellie (CH, Bayonne, France), M Fabbro (Centre Val d'Aurelle Paul Lamarque, Montpellier, France), F Fauchon (Centre de Cobaltothérapie, Nice, France), T Faillot (Hôpital du val de Grâce, Paris, France), P François (Clinique Velpeau, Tours, France), M Frenay (Centre Antoine Lacassagne, Nice, France), G Ganem (Clinique Victor Hugo, Le Mans, France), P Girard-Madoux (Le Forum, Villefranche-sur-Saône, France), C Haglon-Duchemin (Saint Maur des Fosses, France), C Haie-Meder (Institut Gustave Roussy, Villejuif, France), D Hey Mans (Centre Saint Michel, La Rochelle, France), J Hildebrand (Hôpital Erasme, Brussels, Belgium), C Hommet (CHU, Tours, France), J Honnorat (Hôpital Neurocardiologique Pierre Wertheimer, Bron, France), JP Jacquin (Saint Etienne, France), E Jadaud (Centre Paul Papin, Angers, France), M Jan (CHU, Tours, France), A Jouvet (Hôpital Neurocardiologique Pierre Wertheimer, Bron, France), O Joyeux (CH, Valence, France), M Kalamaride (Hôpital Beaujon, Clichy, France), G Kantor (Institut Bergonié Bordeaux, France), B Kin (Clinique Francheville, Périgueux, France), M Kujas (CHU Pitié-Salpêtrière, Paris, France), C Lapras (Clinique du Lac et d’Argonnay, Argonnay, France), DE La Roche (Clinique Mutualiste, Saint Etienne, France), D Larregain Fournier (Hôpital de Bayonne, Bayonne, France), C Lebrun-Frenay, (Hôpital Pasteur, Nice, France), O Lefloch (CHU, Tours, France), R Leloup (CHR, Orléans, France), MH Lemaître (CH, Roanne, France), F Lapierre (CHU, Poitiers, France), T Lesimple (Centre Eugène Marquis, Rennes, France), M Levy-Soussan (CHU Pitié-Salpêtrière, Paris, France), E Lioret (CHU, Tours, France), M Lopes (CHU Pitié-Salpêtrière, Paris, France), C Martin (CH, Annecy, France), O Maton (Clinique Francheville, Perigueux, France), F.Maugière (Hôpital Neurocardiologique Pierre Wertheimer, Bron, France), JF Meder (CH Sainte Anne, Paris, France), J Mikol (Hôpital Lariboisière, Paris, France), B Mogodin (CHG. Montélimar, France), A Monjour (Hôpital Pasteur, Colmar, France), K Mokhtari (CHU Pitié-Salpêtrière, Paris, France), P Monteil (CHU, Bordeaux, France), F Mornex (Hôpital Lyon Sud, Pierre Bénite, France), G Nöel (Centre de Protonthérapie, Orsay, France), F Parker (CHU Bicêtre, Le Kremlin Bicêtre, France), PY Peaud (CH, Valence, France), F Pene (Hôpital Tenon, Paris, France), M Perrier (CHU, Tours, France), JY Pierga (Institut Curie, Paris, France), J Poirier (CHU Pitié-Salpêtrière, Paris, France), E Raymond (Institut Gustave Roussy, Villejuif, France), M Reisenleiter (CH, Vendôme, France), H Robert (CH, Macon, France), GH Robert (Fondation Ophtalmologique Rothschild, Paris, France), H Roché (Centre Claudius Regaud, Toulouse, France), M Ruchoux (Hôpital Robert Salengro, Lille, France), JH Ruel (CH, Annecy, France), P Ryvlin (Hôpital Neurocardiologique Pierre Wertheimer, Bron, France), JF Savet (CH, Macon, France), J Servan (CH René Dubos, Pontoise, France), A Setiey (CHG, Villefranche sur-Saône, France), JM Simon (CHU Pitié-Salpêtrière, Paris, France), M Sindou (Hôpital Neurocardiologique Pierre Wertheimer, Bron, France), J Stecken (CHR, Orléans, France), L Tallandier (Hôpital Saint Julien, Nancy, France), S Tallibert (CHU Pitié-Salpêtrière, Paris, France), P Thiesse (Centre Léon Bérard, Lyon, France), JM Tourani (CHU, Poitiers, France), N Tubiana (CHU, Limoges, France), B Vallee (Hôpital Neurocardiologique Pierre Wertheimer, Bron, France), C Van den Berghe (Centre Médico-Chirurgical de Bligny, Briis sur Forges, France), L Vieillevigne (Centre Eugène Marquis, Rennes, France), A Vighetto (Hôpital Neurocardiologique Pierre Wertheimer, Bron, France), D Vincent (CH, La Rochelle, France).
